# Retraction Note: Down-regulated lncRNA DLX6-AS1 inhibits tumorigenesis through STAT3 signaling pathway by suppressing CADM1 promoter methylation in liver cancer stem cells

**DOI:** 10.1186/s13046-022-02347-9

**Published:** 2022-04-01

**Authors:** Dong-Mei Wu, Zi-Hui Zheng, Ying-Bo Zhang, Shao-Hua Fan, Zi-Feng Zhang, Yong-Jian Wang, Yuan-Lin Zheng, Jun Lu

**Affiliations:** 1 Key Laboratory for Biotechnology on Medicinal Plants of Jiangsu Province, School of Life Science, Jiangsu Normal University, Xuzhou, 221116 Jiangsu Province, People’s Republic of China; 2 College of Health Sciences, Jiangsu Normal University, Xuzhou, 221116 Jiangsu Province, People’s Republic of China; 3 State Key Laboratory Cultivation Base For TCM Quality and Efficacy, School of Medicine and Life Sciences, Nanjing University of Chinese Medicine, Nanjing, 210023 People’s Republic of China; 4 Department of Pathology, Qiqihar Medical University, Qiqihar, 161006 People’s Republic of China

**Retraction Note: J Exp Clin Cancer Res 38, 237 (2019)**

**https://doi.org/10.1186/s13046-019-1239-3**

The Editor-in-Chief has retracted this article. After publication, concerns were raised regarding the data presented in the article. Specifically:

- In Fig. 4j, the western blot bands representing two different cell lines appear highly similar.
- In Figs. 4n, 5c, 6b and 6c, the western blot backgrounds appear inconsistent.
- In Fig. 6d, the images representing Huh7-Blank and HepG2-NC groups appear to contain overlap.

Additionally, the L02 and SMMC-7721 cell lines used in Fig. 1c have been reported to be contaminated with HeLa cells. The authors have been unable to address the above concerns and have stated that these data were obtained from a third party. Furthermore, the authors have been unable to provide evidence of the Ethics Committee approval for the animal study.

The Editor-in-Chief therefore no longer has confidence in the data presented and the conclusions of the article.

Dong-Mei Wu, Zi-Hui Zheng, Shao-Hua Fan, Zi-Feng Zhang, Yong-Jian Wang, Yuan-Lin Zheng and Jun Lu have not explicitly stated whether they agree to this retraction notice. Ying-Bo Zhang has not responded to any correspondence from the editor or publisher about this retraction.

